# Correlation and Interchangeability of Venous and Capillary Blood Gases in Non-Critically Ill Neonates

**DOI:** 10.3389/fped.2018.00089

**Published:** 2018-04-12

**Authors:** Ratna N. G. B. Tan, Steffen C. Pauws, Evelyne van Loon, Vivanne E. H. J. Smits, Enrico Lopriore, Arjan B. te Pas

**Affiliations:** ^1^Division of Neonatology, Department of Paediatrics, Leiden University Medical Center, Leiden, Netherlands; ^2^Tilburg Center for Cognition and Communication, Tilburg University, Tilburg, Netherlands

**Keywords:** blood gases, neonates, venous blood gas, capillary blood gas, interchangeability

## Abstract

**Background:**

Venous blood gas (VBG) is frequently used in the neonatal unit as alternative for capillary blood gas (CBG). However, studies reporting correlation are conflicting and data on interchangeability in neonates are lacking.

**Objective:**

We investigated the correlation and interchangeability of the components between VBG and CBG in infants admitted to the neonatal intensive care unit.

**Methods:**

In a prospective study in the neonatal unit in Leiden University Medical Center (Netherlands), simultaneously VBG and CBG were withdrawn in neonates when both venous puncture and intravenous access as blood gas monitoring was indicated. From each blood gas analysis, a Pearson correlation, intraclass correlation, and Bland–Altman analysis was performed. Clinically acceptable difference for each blood gas value was defined up-front by means of an absolute difference: pH ± 0.05; partial pressure of carbon dioxide (pCO_2_) (±0.67 kPa = 5 mmHg); partial pressure of oxygen (pO_2_) (±0.67 kPa = 5 mmHg); base excess ± 3 mmol/l; and bicarbonate (HCO_3_^−^) ± 3 mmol/l.

**Results:**

In 93 patients [median gestational age 31 (IQR 29–34) weeks], 193 paired samples of VBG and CBG were collected. The Pearson correlation between VBG and CBG was very strong for pH (*r* = 0.79; *P* < 0.001), BE (*r* = 0.90; *P* < 0.001) and bicarbonate (*r* = 0.87; *P* < 0.001); strong for pCO_2_ (*r* = 0.68; *P* < 0.001); and moderate for pO_2_ (*r* = 0.31; *P* < 0.001). The percentage of the interchangeability within our acceptable absolute difference for pH was 88%, pCO_2_ 72%, pO_2_ 55%, BE 90%, and bicarbonate 94%.

**Conclusion:**

VBG and CBG in neonates are well correlated and mostly interchangeable, except for pO_2_.

## Introduction

Patients admitted to the neonatal intensive care unit (NICU) frequently need monitoring of gas exchange and oxygenation (1). Blood gas analysis from an arterial blood gas (ABG) is the gold standard, predominantly withdrawn from an indwelling arterial catheter with continuous heparin solution to prevent clotting (1). However, it can be difficult to obtain an arterial line (1) and serious complications could occur (2). As an alternative for ABG, capillary blood gas (CBG) is accepted (3–13).

According to our recent survey, most neonatologists use venous blood gas (VBG) as an alternative for CBG (14), as venous blood is frequent available due to venepuncture or insertion of intravascular access. Studies performed in pediatric patients demonstrated a good correlation between ABG, CBG, and VBG, which is not a surprising finding (4–7, 15–18). However, to know whether using VBG for blood gas monitoring is a good alternative for CBG, interchangeability would need to be tested. A Bland Altman analysis would then be the best method to test interchangeability (18). In pediatric patients, small studies demonstrated a good correlation and interchangeability in pH, base excess, and bicarbonate (HCO_3_^−^) between CBG and VBG, but the results in pCO_2_ were conflicting (6–9, 15). However, in neonates there are very little data available in the use of VBG as alternative for CBG and the interchangeability has never been quantified. Mc Gillivray showed that clinical decision making was not different when venous or CBG is used (18).

To test the hypothesis that VBG is a good substitute for CBG monitoring for metabolic state, oxygenation, and gas exchange, we determined the correlation and interchangeability between VBG and CBG in neonates admitted to our NICU.

## Materials and Methods

**Figure 1:**
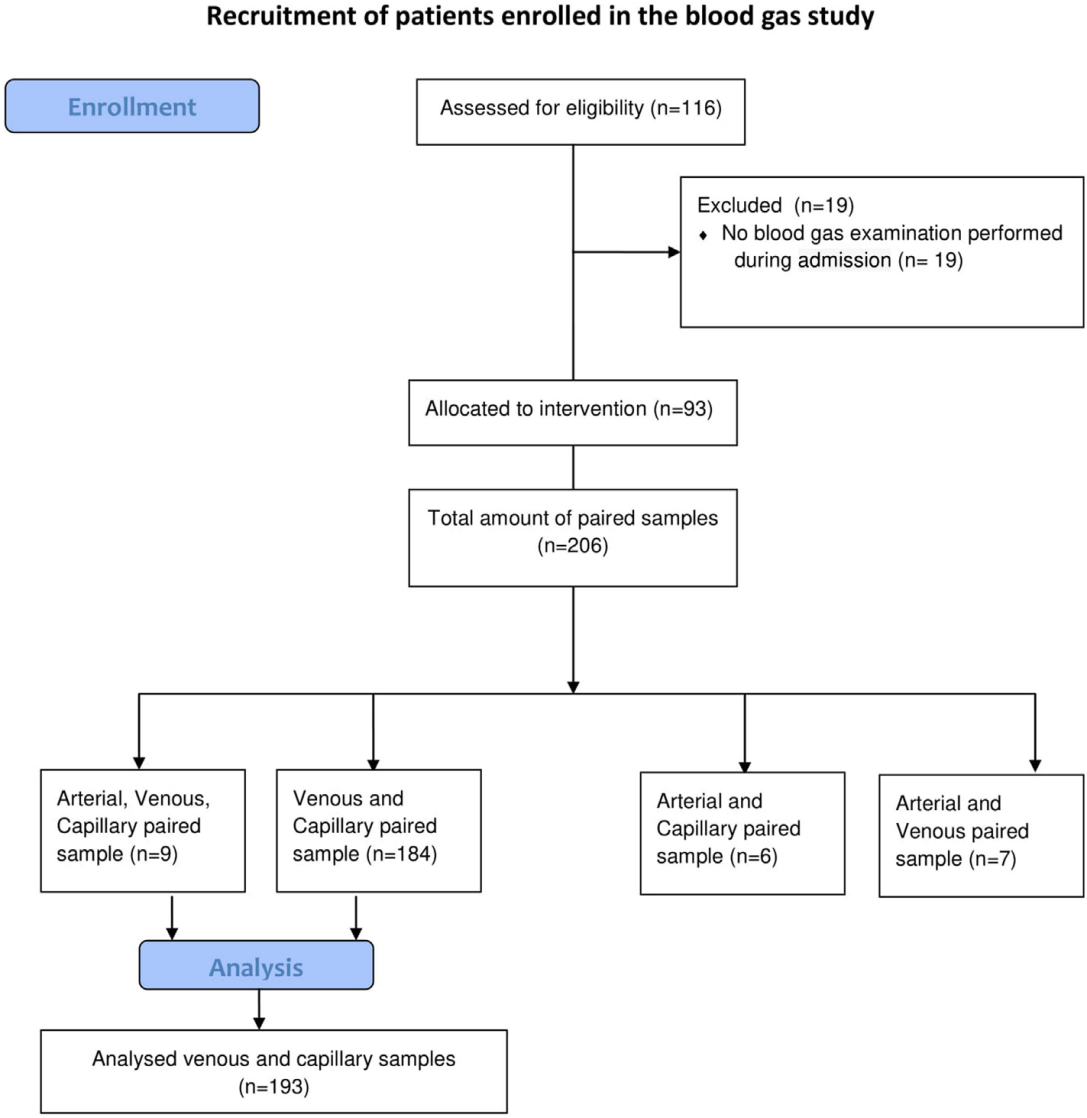
Recruitment of patients enrolled in the blood gas study.

A prospective single center measurement method comparison study was performed from February 2013 until July 2015 in the tertiary NICU in Leiden University Medical Center (Netherlands). All patients needing blood gas analysis, were eligible and parents were asked for informed consent by a member of the medical staff. No incentives were offered (see Figure 1). VBG and CBG were simultaneously withdrawn in neonates, when a venous puncture was performed or intravenous access was inserted, and blood gas monitoring was indicated. All punctures were performed by two caregivers and executed under optimal circumstances; oral sucrose (0.1 ml sucrose 24%) was given prior to puncture and during the procedure comfort was given by the nurse. To minimize the burden per patient, we limited the amount of blood gases per patient to a maximum of 3 paired samples. CBG was withdrawn according to local protocol, without squeezing, from the lateral/medial heel or finger. We used for preterm under 1,500 *g* a One-step safety lance green (1.8 mm)/BD microtainer Quickheel lancet (0.8 mm depth), and for preterm >1,500 *g* a One-step safety lance blue (2.3 mm)/BD microtainer Quickheel lancet (1 mm depth). The heel lances are only performed at the lateral or medial part of the heel, never at the center, conform the suggestions of Noureldein et al. (19). The temperature, color and capillary refill time of the limb and crying of the neonate at the time of the blood sampling was noted. We also recorded some patients’ characteristics (blood pressure, respiratory support, and inotropic use). A blood gas sample of 60 µl of blood was collected in Siemens healthcare diagnostic pre-heparinized capillary tubes containing 130–200 IU heparin/ml. The collected blood gases were analyzed within 5 min using a Siemens Rapidlab 860 analyzer without co-oximetry available in the nursery.

### Ethical Statement

The study was approved by the Medical Ethical Committee of LUMC.

### Statistics

A Pearson correlation and intraclass coefficient (two-way mixed and absolute agreement) of the different parts of the blood gases were calculated. To determine interchangeability, a Bland–Altman analysis was performed, based on the 95% limits of agreement (LoA), estimated by mean difference (bias) ±2 SD of the differences, that provides a 95% confidence interval of the differences between VBG and CBG are expected to lie. Clinically acceptable difference for each blood gas value was defined up-front by means of an absolute difference: pH (±0.05), pCO_2_ (±0.66 kPa = 5 mmHg), pO_2_ (± 0.66 kPa = 5 mmHg), BE (±3 mmol/l/), and bicarbonate (±3 mmol/l), based on bias of reference values of infant’s blood gas (11, 20) and former studies (5, 6, 17). The oxygen saturation of our blood gasses are estimated values, not measured by co-oximetry, and was not included in our analyses.

All values were checked for normal distribution, proportional bias, and systemic bias, for which a correction was performed when needed. All statistical analysis were performed using IBM SPSS Statistics, version 20.0 and using a significance level of α = 0.05. We used two-sample non-inferiority or superiority calculator to compare two means, using type error rate of α = 5% and power of 80%. The non-inferiority or superiority margin was defined equally to our clinical acceptable difference. For sample size, 158 paired samples was needed, which was based on capillary and VBGs reference values for pCO_2_ [capillary mean (SD) pCO_2_ 5.83 (1.4) kPa, venous mean (SD) pCO_2_ 5.99 (1.4) kPa] (10). In order to evaluate whether patients characteristics or conditions when blood samples were taken influenced the interchangeability of pCO_2_, we performed a Mann–Whitney *U* test, a chi square test, and a mean independent-test where appropriate.

## Results

In total, 193 paired samples of VBG and CBG of 93 neonates were analyzed (patient characteristics: Table 1). The extremities were well circulated when the sample was taken in 181/193 (94%) samples and neonates were crying during 30/193 (16%) samples.

**Table 1:** Patient characteristics.

	All patients = 194
Gestational age in weeks, median (IQR)	31 (29–34)
Weight in g, median (IQR)	1,495 (1,079–1,975)
Postnatal age in days, median (IQR)	7 (3–13)
Female, *n* (%)	90 (47)
Warm and pink extremities, *n* (%)	181 (94)
Venous blood sample from hand/foot, *n* (%)	189 (98)
Capillary blood sample from heel, *n* (%)	191 (99)
Crying during sampling, *n* (%)	30 (16)
Capillary refill time central <3 s, *n* (%)	173 (90)
Capillary refill time peripheral <3 s, *n* (%)	182 (94)
Respiratory support, total, *n* (%)	108 (56)
Invasive ventilation, *n* (%)	27 (14)
Non-invasive ventilation, *n* (%)	63 (32)
Temperature of the skin (*n* = 181), median (IQR)	36.8 (36.5–37.1)
Rectal body temperature (*n* = 142), median (IQR)	37.0 (36.7–37.2)
Use of inotropic, *n* (%)	5 (3)
Hypotension defined as mean blood pressure below gestational age, *n* (%)	5 (3)

The VBG and CBG was significant and very strong positively correlated for pH (*r* = 0.79), BE (*r* = 0.90), and bicarbonate (*r* = 0.87), strong positively correlated for pCO_2_ (*r* = 0.68) and weak positively correlated for pO_2_ (*r* = 0.31) (Table 2). The intraclass coefficient for average measures between VBG and CBG had an almost perfect agreement for pH [intraclass coefficient correlation (ICC) = 0.87], for PCO_2_ (ICC = 0.802); BE (ICC = 0.946), bicarbonate (ICC = 0.928) and fair agreement for pO_2_ (ICC = 0.364) (Table 2). The mean difference and 95% LoA between VBG and CBG was for BE (0.0 mmol/l [−3.5, +3.5]) and for bicarbonate (0.2 mmol/l [−3.9, +4.3]); and after correction for fixed bias for pH (0.00 [−0.08, 0.08]), pCO_2_ (0.0 kPa [−1.8, 1.8]), and pO_2_ (−0.9 kPa [−4.1, 3.3]).

**Table 2:** The Pearson correlation, intraclass coefficient, and Bland–Altman analysis, including limits of agreement (LoA) of the different components of capillary blood gas and venous blood gas.

	Pearson correlation		Intraclass coefficient correlation (ICC) average measures			Bland–Altman analysis				
			
	*r*	*P*-value	ICC	95% CI	*P*-value	MD	SD	95% LoA	Clinical acceptable difference	% within clinical acceptable difference
pH	0.79^a^	0.00	0.87^b^	0.82–0.90^b^	0.00	−0.01	0.40	−0.10, +0.08	±0.05	86
pCO_2_ kPa	0.68	0.00	0.80^b^	0.74–0.85	0.00	0.17	0.94	−1.67, +2.00	±0.67	67
pO_2_ kPa	0.31	0.00	0.364	0.16–0.52	0.00	0.40	1.88	−4.09, +3.29	±0.67	47
BE mmol/l	0.9^a^	0.00	0.95^b^	0.93–0.96^b^	0.00	−0.01	1.80	−3.53, +3.51	±3	90
HCO_3_- mmol/l	0.87^a^	0.00	0.93^b^	0.91–0.95^b^	0.00	0.21	2.10	−3.92, +4.34	±3	94
Corrected pH Bias +0.01						0.00	0.04	−0.08, +0.08	±0.05	88
Corrected pCO_2_ kPa bias −0.166						0.00	0.94	−1.83, +1.83	±0.67	72
Corrected pO_2_ kPa bias +0.399						−0.92	1.60	−4.05, +2.21	±0.67	55

*^a^Pearson correlation: very strong correlation (*r* > 0.7)*.

*^b^ICC: almost perfect agreement (ICC > 0.8)*.

*CI, confidence interval; MD, mean difference*.

**Figure 2:**
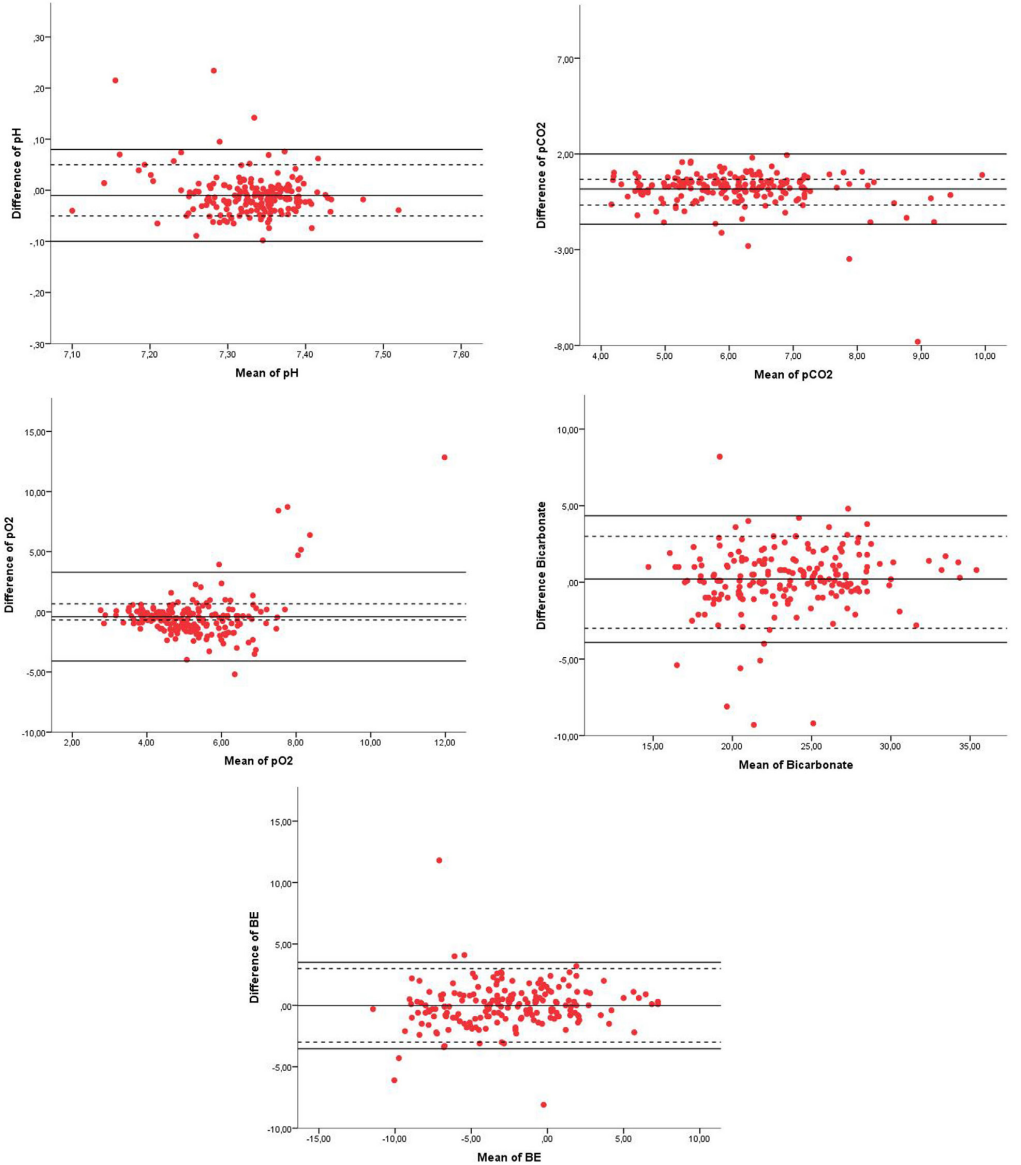
Bland–Altman plots of pH, pCO_2_, pO_2_, bicarbonate, and BE of venous blood gas (VBG) and capillary blood gas (CBG). Legend of the Bland–Altman plots: *X* = mean of pH, pCO_2_ (kpa), pO_2_ (kpa), bicarbonate (mmol/1), or BE (mmol/1), *Y* = difference of the parameter of pH, pCO_2_ (kpa), pO_2_ (kpa), bicarbonate (mmol/1), or BE (mmol/1), *red dots* = measurement of each paired sample of VBG and CBG, *solid black line* = 95% confidence interval limits, and *dotted line* = clinical acceptable absolute difference.

From all components of the blood gases, the 95% LoA were not within the range of our clinical acceptable absolute difference (Table 2; Figure 2). The percentage of values within our acceptable absolute difference was for pH 88%, pCO_2_ 72%, pO_2_ 55%, BE 90%, and bicarbonate 94% (Table 2). Analysis of patients characteristics and conditions when blood samples were taken and showed that only crying was significantly higher in the group with pCO_2_ outside when compared to the group within the acceptable difference for pCO_2_; 25 vs. 11%; *P* = 0.02.

## Discussion

Our study added a very strong linear correlation of pH, pCO_2_, BE, and bicarbonate, with an almost perfect agreement based on ICC correlation between VBG and CBG, except for pO_2_. In addition, the components of VBG were interchangeable with CBG for the majority of the samples, except for pO_2_. This implies that VBGs can be used to monitor gas exchange and metabolic state of neonates. Separately CBG should be limited to the minimum, especially now recent study added the safety concerns of CBG in neonates (10, 19). For evaluating oxygenation, ABGs are already considered golden standard.

The correlation of pH, pO_2_, BE, and bicarbonate between VBG and CBG was comparable to previous studies in neonates and pediatric patients (4, 6, 15–17). By contrast, while we observed a good correlation of pCO_2_ between VBG and CBG, this correlation varied between studies (4, 6, 15, 16, 21). Some studies described that correlation and agreement of pCO_2_ is poor in very sick pediatric patients (6, 15). Bilan et al. recognized a good validity and clinical agreement based on kappa statistics for acid–base imbalance (pH, pCO_2_, BE) for most intensive care pediatric and neonatal patients but not when there was congestive heart failure or shock (15). Neonates with shock were not included in our study as most of these had an arterial catheter inserted for monitoring.

We reported a high degree of interchangeability for the different components of blood gas. The venous pH, BE, and bicarbonate were for more than 88% interchangeable and therefore a reliable substitute for CBG, similar to previous results of pediatric and adults studies (4, 6, 17, 22, 23). However, the interchangeability of pCO_2_ (72%) was lower in our study when compared to the 90% in pediatric and adults studies (4, 17, 23). It is difficult to explain this difference in pCO_2_ while the other parts of the measurements were similar. Perhaps technical errors (e.g., air bubbles) could have occurred, as this has more influence on the pCO_2_ than on the metabolic component. Tachypnea due to pain and discomfort during venepuncture could also have influenced the pCO_2_, as a large proportion of our cohort was not sedated, while most children and adults in the other studies were probably sedated. Sampling from a venous central line (4, 22, 23) is painless and creates a more constant circumstance. Indeed, our analysis demonstrated a significantly higher percentage crying in the patient group with pCO_2_ outside the clinical acceptable difference. It is also possible that the puncture was more difficult in a crying and uncomfortable neonate, and increased the chance for technical errors. For analysis, we assumed that all samples per individual were independent separate measurements as they were taken spread in time at three different moments during the NICU admission and not performed subsequently after each other (24).

The good correlation of pCO_2_ and not for pO_2_ can be explained by the oxygen–hemoglobin dissociation curve and carbon oxide equilibrium curve. A previous study in adults reported that pO_2_ differ more in arterial, capillary and venous values than pCO_2_ (18). This can be explained by the S-shaped oxygen–hemoglobin dissociation curve, where different types of blood samples are reflected in different regions of the curve: venous blood in the steep region, whereas arterial blood in the plateau region. Therefore, a slight change affects the pO_2_ more in venous blood compared to arterial or arterialized capillary blood. In contrast to pO_2_, pCO_2_ is described in a linear carbon dioxide equilibrium curve for both arterial as venous blood. The carrying capacity of blood CO_2_ is much greater than for O_2_, and blood can load and unload large amounts of CO_2_ with small difference in gas tension, for the regulation of gas exchange and acid–base balance (18). Therefore, the observed pCO_2_ values between arterialized capillary and venous are comparable, and the pO_2_ not.

## Conclusion

Our findings support the use of VBG in neonates for metabolic evaluation and monitoring gas exchange, but not for monitoring oxygenation. In most occasions, the blood gasses are interchangeable; therefore when a venepuncture is performed, a VBG can be withdrawn simultaneously to limit the burden for patient.

## Ethics Statement

Medical ethical committee of Leiden Medical University approved this study, including the recruiting method and asking parents or caregivers for consent by trained caregivers. All the participants from whom we collected the data, a written consent is available.

## Author Contributions

RT carried out the data collection, data analyses, and initially wrote, revised, reviewed, and approved the manuscript. AP conceptualized and designed the study, and reviewed, revised, and approved the manuscript. SP critically reviewed the data analyses, and reviewed and approved the final manuscript. EL, VS, and EL critically reviewed and approved the final manuscript.

## Conflict of Interest Statement

The authors declare that the research was conducted in the absence of any commercial or financial relationships that could be construed as a potential conflict of interest.

## References

[1] Goldsmith JP Karotkin EH Assisted Ventilation of the Neonate. 4th ed St. Louis: Elsevier Saunders (2003).

[2] Hermansen MC Hermansen MG Intravascular catheter complications in the neonatal intensive care unit. Clin Perinatol (2005) 32(1):141–56, vii. 10.1016/j.clp.2004.11.005 15777826

[3] McLain BI Evans J Dear PR Comparison of capillary and arterial blood gas measurements in neonates. Arch Dis Child (1988) 63(7 Spec No):743–7. 10.1136/adc.63.7_Spec_No.743 3137897 PMC1590118

[4] Yildizdas D Yapicioglu H Yilmaz HL Sertdemir Y. Correlation of simultaneously obtained capillary, venous, and arterial blood gases of patients in a paediatric intensive care unit. Arch Dis Child (2004) 89(2):176–80. 10.1136/adc.2002.016261 14736638 PMC1719810

[5] Harrison AM Lynch JM Dean JM Witte MK. Comparison of simultaneously obtained arterial and capillary blood gases in pediatric intensive care unit patients. Crit Care Med (1997) 25(11):1904–8. 10.1097/00003246-199711000-00032 9366777

[6] Kirubakaran C Gnananayagam JE Sundaravalli EK. Comparison of blood gas values in arterial and venous blood. Indian J Pediatr (2003) 70(10):781–5. 10.1007/BF02723794 14649471

[7] MacRae DJ Palavradji D Comparison between arterial, capillary and venous acid-base measurements in the newborn infant. J Obstet Gynaecol Br Commonw (1966) 73(5):761–5. 10.1111/j.1471-0528.1966.tb06080.x 5919095

[8] Stamm SJ Reliability of capillary blood for the measurement of pO_2_ and O_2_ saturation. Dis Chest (1967) 52(2):191–4. 10.1378/chest.52.2.191 6038777

[9] Glasgow JF Flynn DM Swyer PR A comparison of descending arotic and “arterialized” capillary blood in the sick newborn. Can Med Assoc J (1972) 106(6):660–2. 5062428 PMC1940510

[10] Kokholm G Simultaneous measurements of blood pH, pCO_2_, pO_2_ and concentrations of hemoglobin and its derivates – a multicenter study. Scand J Clin Lab Invest Suppl (1990) 203:75–86. 10.3109/00365519009087494 2128562

[11] Cousineau J Anctil S Carceller A Gonthier M Delvin EE. Neonate capillary blood gas reference values. Clin Biochem (2005) 38(10):905–7. 10.1016/j.clinbiochem.2005.07.006 16109393

[12] Zavorsky GS Cao J Mayo NE Gabbay R Murias JM. Arterial versus capillary blood gases: a meta-analysis. Respir Physiol Neurobiol (2007) 155(3):268–79. 10.1016/j.resp.2006.07.002 16919507

[13] Escalante-Kanashiro R Tantalean-Da-Fieno J Capillary blood gases in a pediatric intensive care unit. Crit Care Med (2000) 28(1):224–6. 10.1097/00003246-200001000-00037 10667527

[14] Tan RN Mulder EE Lopriore E Te Pas AB. Monitoring oxygenation and gas exchange in neonatal intensive care units: current practice in the Netherlands. Front Pediatr (2015) 3:94. 10.3389/fped.2015.00094 26579504 PMC4630576

[15] Bilan N Behbahan AG Khosroshahi AJ. Validity of venous blood gas analysis for diagnosis of acid-base imbalance in children admitted to pediatric intensive care unit. World J Pediatr (2008) 4(2):114–7. 10.1007/s12519-008-0022-x 18661766

[16] McGillivray D Ducharme FM Charron Y Mattimoe C Treherne S. Clinical decision-making based on venous versus capillary blood gas values in the well-perfused child. Ann Emerg Med (1999) 34(1):58–63. 10.1016/S0196-0644(99)70272-6 10381995

[17] Tobias JD Connors D Strauser L Johnson T. Continuous pH and pCO_2_ monitoring during respiratory failure in children with the paratrend 7 inserted into the peripheral venous system. J Pediatr (2000) 136(5):623–7. 10.1067/mpd.2000.104293 10802494

[18] Rhoades RA Tanner GA Medical Physiology. Gas Transfer and Transport. USA: Little Brown (1995). p. 386–400.

[19] Noureldein M Gowda H Is it safe to use the centre of the heel for obtaining capillary blood samples in neonates? Arch Dis Child (2018) 103:401–4. 10.1136/archdischild-2017-314214 29348114

[20] Brouillette RT Waxman DH. Evaluation of the newborn’s blood gas status. National academy of clinical biochemistry. Clin Chem (1997) 43(1):215–21. 8990256

[21] Bland JM Altman DG. Measuring agreement in method comparison studies. Stat Methods Med Res (1999) 8(2):135–60. 10.1191/096228099673819272 10501650

[22] Treger R Pirouz S Kamangar N Corry D Agreement between central venous and arterial blood gas measurements in the intensive care unit. Clin J Am Soc Nephrol (2010) 5(3):390–4. 10.2215/CJN.00330109 20019117 PMC2827573

[23] Tobias JD. Transcutaneous carbon dioxide monitoring in infants and children. Paediatr Anaesth (2009) 19(5):434–44. 10.1111/j.1460-9592.2009.02930.x 19236597

[24] Bland JM Altman DG. Agreement between methods of measurement with multiple observations per individual. J Biopharm Stat (2007) 17(4):571–82. 10.1080/10543400701329422 17613642

